# Predictive model for HBsAg clearance rate in chronic hepatitis B patients treated with pegylated interferon α-2b for 48 weeks

**DOI:** 10.1007/s12072-024-10764-5

**Published:** 2024-12-19

**Authors:** Zhili Tan, Nan Kong, Qiran Zhang, Xiaohong Gao, Jia Shang, Jiawei Geng, Ruirui You, Tao Wang, Ying Guo, Xiaoping Wu, Wenhong Zhang, Lihong Qu, Fengdi Zhang

**Affiliations:** 1 Department of Infectious Diseases, School of Medicine, Shanghai East Hospital, Tongji University, Shanghai, China; 2 Department of Infectious Diseases, Shanghai Key Laboratory of Infectious Diseases and Biosafety Emergency Response, National Medical Center for Infectious Diseases, Huashan Hospital, Shanghai Medical College, Fudan University, Shanghai, China; 3 Department of Infectious Diseases, Yanan University Affiliated Hospital, Yan’an, Shaanxi China; 4 Department of Infectious Disease and Hepatic Disease, Henan Provincial People’s Hospital, Henan, China; 5 Department of Infectious Disease and Hepatic Disease, First People’s Hospital of Yunnan Province, Affiliated Hospital of Kunming University of Science and Technology, Kunming, Yunnan China; 6 Deparment of Hepatology, The Third People’s Hospital of Taiyuan, Taiyuan, China; 7 Department of Infectious Diseases, the First Affiliated Hospital of Nanchang University, Nanchang, China

**Keywords:** Chronic Hepatitis B (CHB), HBsAg clearance, Predictive model, Logistic regression, Clinical prognosis

## Abstract

**Background and Aims:**

Chronic hepatitis B (CHB) is a major global health concern. This study aims to investigate the factors influencing hepatitis B surface antigen (HBsAg) clearance in CHB patients treated with pegylated interferon α-2b (Peg-IFNα-2b) for 48 weeks and to establish a predictive model.

**Methods:**

This analysis is based on the “OASIS” project, a prospective real-world multicenter study in China. We included CHB patients who completed 48 weeks of Peg-IFNα-2b treatment. Patients were randomly assigned to a training set and a validation set in a ratio of approximately 4:1 by spss 26.0, and were divided into clearance and non-clearance groups based on HBsAg status at 48 weeks. Clinical data were analyzed using SPSS 26.0, employing chi-square tests for categorical data and Mann–Whitney *U* tests for continuous variables. Significant factors (*p* < 0.05) were incorporated into a binary logistic regression model to identify independent predictors of HBsAg clearance. The predictive model’s performance was evaluated using ROC curve analysis.

**Results:**

We included 868 subjects, divided into the clearance group (187 cases) and the non-clearance group (681 cases). They were randomly assigned to a training set (702 cases) and a validation set (166 cases). Key predictors included female gender (OR = 1.879), lower baseline HBsAg levels (OR = 0.371), and cirrhosis (OR = 0.438). The final predictive model was:

Logit(P) = 0.92 + Gender (Female) * 0.66 - HBsAg (log) * 0.96 - Cirrhosis * 0.88. ROC analysis showed an AUC of 0.80 for the training set and 0.82 for the validation set, indicating good predictive performance.

**Conclusion:**

Gender, baseline HBsAg levels, and cirrhosis are significant predictors of HBsAg clearance in CHB patients after 48 weeks of Peg-IFNα-2b therapy. The developed predictive model demonstrates high accuracy and potential clinical utility**.**

**Supplementary Information:**

The online version contains supplementary material available at 10.1007/s12072-024-10764-5.

## Introduction

Chronic Hepatitis B (CHB) is a persistent inflammatory liver disease caused by continuous infection with Hepatitis B Virus (HBV). Epidemiological data estimate that the prevalence of HBsAg in the general population in China was 6.1% in 2016, with 86 million cases of chronic HBV infection [1]. Hepatocellular carcinoma (HCC) is the 6th most common cancer globally and the 3rd leading cause of cancer-related deaths [2]. CHB is a major cause of HCC, with approximately 84% of liver cancer in China being CHB-related. CHB imposes a significant disease burden in China and is a major public health challenge. The goal of CHB treatment is to reduce the risk of HCC and decompensated cirrhosis, as well as to improve long-term prognosis.

Currently, the first-line medications for treating CHB are Peg-IFNα−2b and nucleos(t)ide analogues (NAs). During HBV infection and replication, a specific structure called covalently closed circular (ccc) DNA serves as a template for HBV transcription and replication, and as a reservoir for viral genes. This structure is key to the difficulty in curing CHB [3]. NAs primarily inhibit virus replication by blocking the reverse transcription process. However, due to their inability to directly suppress cccDNA transcriptional activity, there is a high rate of relapse after withdrawal, necessitating long-term drug maintenance [4, 5]. Peg-IFNα−2b is currently recognized as the only antiviral medication that can increase the rate of functional cure for CHB. In addition to inhibiting virus replication, it also impacts cccDNA transcriptional inhibition, degradation, and clearance of HBV-infected cells, which is more conducive to virus clearance [6–8]. Previous studies have observed different HBsAg seroconversion rates at 48 weeks of Peg-IFNα−2b monotherapy depending on the selected population [9, 10].

This study evaluates and models the factors affecting HBsAg clearance using data from the "OASIS" project, with the aim of establishing a scientific probability prediction model for seroconversion to guide the clinical selection of optimal CHB treatment options.

## Methods

### Subjects

The “OASIS” project (NCT04896255), initiated by the China Hepatitis Prevention Foundation, is a multicenter, prospective real-world study aimed at reducing the incidence of liver cancer in hepatitis B patients. The project includes treatment-naïve, IFN-treated, and NA-treated CHB patients from 32 provinces across China. Subjects receive either Peg-IFNα−2b-based treatment (Peg-IFNα−2b monotherapy or in combination with NAs) or NAs monotherapy and are followed up for 5 years.

This study included participants from the “Oasis Project” who met the specific inclusion criteria: 1) chronic HBV infection (HBsAg positive for more than 6 months, or HBsAg positive for less than 6 months but liver biopsy within 1 year confirmed characteristics of CHB and ruled out other liver diseases); 2) aged 18 to 80, of any gender; 3) patients using Peg-IFNα−2b for antiviral therapy; and 4) completed 48 weeks of follow-up.

### Study procedures

**Fig. 1:**
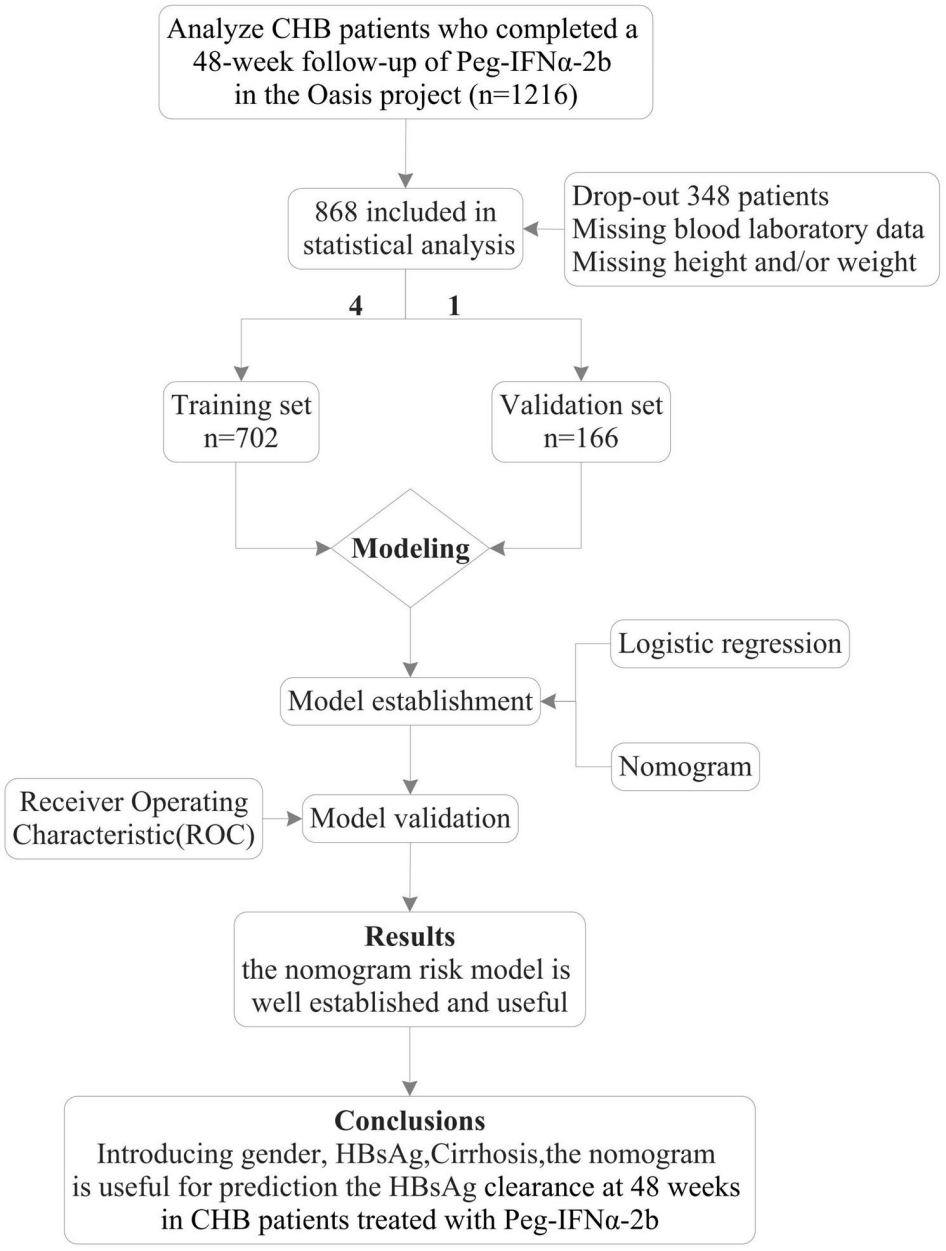
Flow diagram of study design

Among the initially identified 1216 patients with CHB, 348 were excluded from the final analysis due to loss of follow-up or missing clinical trial data. Therefore, the final evaluated patient cohort consisted of 868 patients with CHB. The study design flow chart is shown in Fig. 1.

Firstly, 868 patients were included according to the entry criteria, and then randomly divided into two datasets in a ratio of approximately 4:1, namely the training set (702 patients) and the validation set (166 patients). In the training set, patients were divided into a clearance group (152 patients) and a non-clearance group (550 patients) based on HBsAg status at 48 weeks. We used univariate and multivariate regression analysis to determine the independent influencing factors of HBsAg clearance. Then constructed a predictive function model and drawn nomograms based on these factors.

### Statistical analyses

Statistical analysis was conducted using SPSS 26.0 (IBM, Armonk, NY, USA) and R 4.3.3 (R Foundation for Statistical Computing, Vienna, Austria). Categorical data were presented as frequencies and percentages and analyzed using the chi-square test. Continuous variables were presented as median with interquartile range (Q1, Q3) and analyzed using the Mann–Whitney *U* test. A binary logistic regression model (stepwise forward selection) was utilized to establish the predictive model. The ROC curve was used to analyse the model’s discrimination, and the calibration curve and Hosmer–Lemeshow test were used to assess the calibration of the prediction model. Two-tailed tests with a confidence interval of 95% and statistical significance set at *p* < 0.05 were applied for all analyses.

## Results

### Characteristics of the study

This study included 868 CHB patients, among whom 187 cases (21.5%) achieved clearance, and 681 cases (78.5%) did not. Differential analysis revealed statistically significant differences (*p* < 0.05) in gender, BMI, baseline HBsAg, baseline HBeAg, baseline HBV DNA, ALT, AST, GGT, albumin, AFP, and HGB between the two groups, as shown in Table 1. Table 2 shows the detailed baseline characteristics of the training set (702 patients) and the validation set (166 patients). There were no statistically significant differences between the training set and the validation set in terms of various variables (*p* > 0.05), indicating a matching balance.

**Table 1:** Baseline characteristics of all participants

Variables	Total patient (*n* = 868)	Clearance group (*n* = 187)	Non-clearance group (*n* = 681)	*p*
Age (years)	38 (32,45)	38 (32,46)	38 (32,45)	0.396
Male gender, *n* (%)	633 (72.90%)	122 (65.20%)	511 (75.00%)	0.008
BMI (kg/m^2^)	23.66 (21.45,25.40)	23.12 (20.76,25.06)	23.77 (21.63,25.50)	0.030
Cirrhosis, *n* (%)	115 (13.20%)	17 (9.10%)	98 (14.40%)	0.058
Family history of hepatitis B, *n* (%)	298 (34.30%)	54 (28.90%)	244 (35.80%)	0.076
Past history of therapy, *n* (%)				0.489
None	39 (4.50%)	7 (3.70%)	32 (4.70%)	
NA	290 (33.40%)	57 (30.50%)	233 (34.20%)	
IFN	539 (62.10%)	123 (65.80%)	416 (61.10%)	
HBsAg (log_10_ IU/ml)	2.88 (2.02,3.50)	1.76 (0.76,2.59)	3.13 (2.39,3.62)	< 0.001
HBeAg positive, *n* (%)	279 (32.10%)	29 (15.50%)	250 (36.70%)	< 0.001
HBV DNA (IU/ml), *n* (%)				< 0.001
≤ 2000	602 (69.40%)	160 (85.60%)	442 (64.90%)	
> 2000	266 (30.60%)	27 (14.40%)	239 (35.10%)	
ALT (U/L)	30.00 (20.00,51.00)	25.40 (16.50,36.10)	32.00 (21.00,54.45)	< 0.001
AST (U/L)	26.00 (21.00,31.15)	23.70 (19.50,29.40)	26.2 0(21.00,38.66)	< 0.001
TBIL (μmol/L)	15.11 (11.40,19.99)	15.10 (11.41,20.40)	15.12 (11.36,19.92)	0.722
GGT (U/ L)	23.48 (16.00,36.40)	19.00 (13.00,30.00)	24 .00(17.00,38.00)	< 0.001
Albumin (g/ L)	45.00 (43.00,47.30)	46.00 (43.40,47.80)	45.00 (43.00,47.10)	0.035
AFP (ng/ml)	2.87 (2.00,4.07)	2.54 (1.73,3.76)	2.93 (2.09,4.30)	< 0.001
WBC (10^9^/ L)	5.37 (4.39,6.45)	5.46 (4.46,6.40)	5.34 (4.39,6.45)	0.695
HGB (g/ L)	153 (140,163)	150 (135,160)	154 (141,164)	0.007
PLT (10^9^/ L)	197 .00(159.00,237.00)	210.00 (156.00,248.00)	196.00 (160.00,232.50)	0.087

Continuous variables in the table, including Age, BMI, HBsAg (log_10_), ALT, AST, TBIL, GGT, Albumin, AFP, WBC, HGB, and PLT, were presented as median (25th percentile, 75th percentile) due to their non-normal distribution

**Table 2:** Baseline characteristics of patients in Training Set and Validation Set

Variables	Training set (*n* = 702)	Validation set (*n* = 166)	*p*
Age (years)	38 (32,46)	37 (32,45)	0.853
Male gender, *n* (%)	516 (73.50%)	117 (70.50%)	0.431
BMI (kg/m^2^)	23.66 (21.62,25.47)	23.67 (20.76,25.34)	0.387
Cirrhosis, *n* (%)	97 (13.80%)	18 (10.80%)	0.309
Family history of hepatitis B, *n* (%)	244 (34.80%)	54 (32.50%)	0.587
Past history of therapy, *n* (%)			0.490
None	34 (4.80%)	5 (3.00%)	
NA	237 (33.80%)	53 (31.90%)	
IFN	431 (61.40%)	108 (65.10%)	
HBsAg (log_10_ IU/ml)	2.89 (2.01,3.49)	2.85 (2.07,3.54)	0.957
HBeAg positive, *n* (%)	230 (32.80%)	49 (29.50%)	0.421
HBV DNA (IU/ml), *n* (%)			0.251
≤ 2000	493 (70.20%)	109 (65.70%)	
> 2000	209 (29.80%)	57 (34.30%)	
ALT (U/L)	29.00(20.00,49.00)	32.05 (19.60,62.50)	0.154
AST (U/L)	25.65 (21.00,34.00)	27.65 (20.63,40.25)	0.246
TBIL (μmol/L)	15.13 (11.50,20.02)	15.00 (11.10,20.03)	0.361
GGT (U/ L)	23.85 (15.15,36.31)	23.00 (17.00,36.55)	0.536
Albumin (g/ L)	45.20 (43.00,47.40)	44.90 (43.00,47.00)	0.271
AFP (ng/ml)	2.87 (1.98,4.04)	2.93 (2.04,4.19)	0.315
WBC (10^9^/ L)	5.38 (4.48,6.46)	5.19 (4.18,6.30)	0.105
HGB (g/ L)	153.00 (140.00,163.00)	153.00 (138.75,162.00)	0.639
PLT (10^9^/ L)	198.00 (157.75,238.00)	195.00 (163.00,230.25)	0.652

Continuous variables in the table, including Age, BMI, HBsAg (log_10_), ALT, AST, TBIL, GGT, Albumin, AFP, WBC, HGB, and PLT, were presented as median (25th percentile, 75th percentile) due to their non-normal distribution

### Univariate and multifactorial logistic regression analysis

Univariate analysis of associations between variables and the two groups(clearance group and non-clearance group) are shown (Table 3). Based on the univariate logistic regression analyses, the following data were selected for further analysis: gender, BMI, cirrhosis, baseline HBsAg, baseline HBeAg, baseline HBV DNA, ALT, AST, GGT, albumin, AFP, and HGB. Multiple logistic regression was used to verify the independence of these variables after the stepwise selection process. The results indicated that gender, baseline HBsAg level, and cirrhosis were independent factors affecting the clearance of 48-week HBsAg (Table 3): female (OR = 1.94, 95% CI 1.24 ~ 3.05), baseline HBsAg (log_10_ IU/ml) (OR = 0.38, 95% CI 0.32 ~ 0.46), and cirrhosis (OR = 0.42, 95% CI 0.21 ~ 0.83).

**Table 3:** Univariate and multiple logistic regression analysis of factors associated with HBsAg clearance at the end of 48-week Peg-IFNα−2b treatment in training set

Variable	Univariate logistic regression					Multiple logistic regression model				
	B	*p*	OR	95% CI		B	*p*	OR	95% CI	
				Lower	Upper				Lower	Upper
Age	0.01	0.33	1.01	0.99	1.03					
gender, female vs male	0.40	0.04	1.49	1.01	2.20	0.66	< 0.01	1.94	1.24	3.05
BMI	−0.06	0.04	0.94	0.88	1.00					
Cirrhosis, Cirrhosis vs non-Cirrhosis	−0.66	0.04	0.52	0.28	0.96	−0.88	0.01	0.42	0.21	0.83
Family history of hepatitis B, yes vs no	−0.34	0.09	0.71	0.48	1.05					
Past history of therapy										
None vs IFN	−0.17	0.71	0.85	0.36	2.00					
NA vs IFN	−0.30	0.15	0.75	0.50	1.11					
HBsAg (log_10_ IU/ml)	−0.92	< 0.001	0.40	0.33	0.48	−0.96	< 0.01	0.38	0.32	0.46
HBeAg, positive vs negative	−1.11	< 0.001	0.33	0.21	0.53					
HBV DNA, > 2000 vs ≤ 2000	−1.05	< 0.001	0.35	0.22	0.56					
ALT (U/L)	−0.01	0.01	0.99	0.99	1.00					
AST (U/L)	−0.01	0.01	0.99	0.98	1.00					
TBIL (μmol/L)	0.00	0.63	1.00	0.98	1.01					
GGT (U/ L)	−0.01	0.02	0.99	0.98	1.00					
Albumin (g/ L)	0.03	0.26	1.03	0.98	1.08					
AFP (ng/ml)	−0.13	0.01	0.88	0.80	0.96					
WBC (10^9/ L)	0.04	0.46	1.04	0.94	1.15					
HGB (g/L)	0.00	0.41	1.00	0.99	1.01					
PLT (10^9/L)	0.00	0.30	1.00	1.00	1.01					
Constant						0.54	0.01	1.71		

### Predictive model construction

**Fig. 2:**
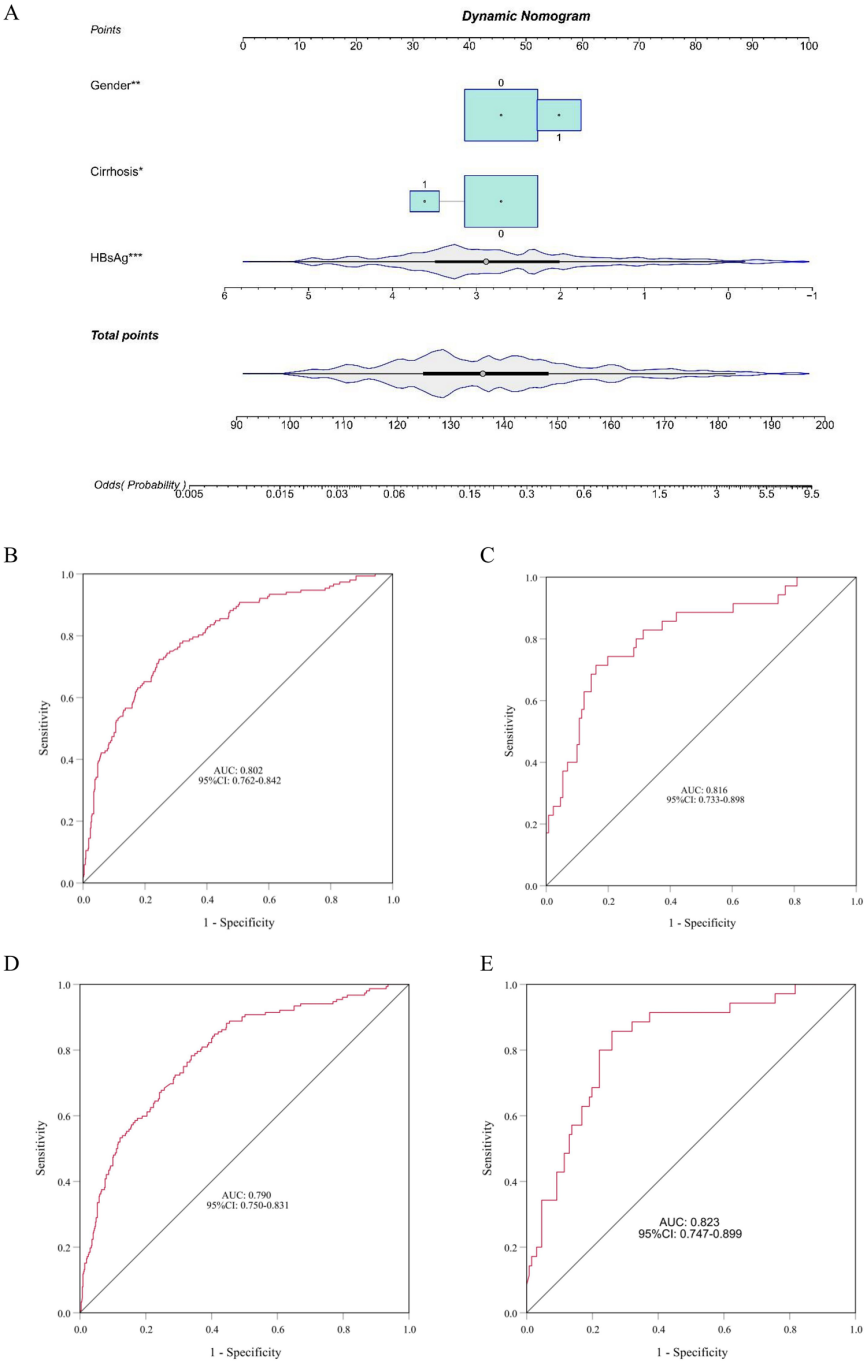
The Nomogram (predictive function models) and ROC curves (predictive function models and HBsAg) (**A**) Nomogram for predicting 48-week HBsAg clearance. Influencing factors of gender, HBsAg (log_10_), cirrhosis for nomogram prediction model. **B**, **C** The ROC curves of the predictive function models for the training set (**B**) and validation set (**C**). **D**, **E** The ROC curves of HBsAg for the training set (**D**) and validation set (**E**)

After 48 weeks of Peg-IFNα−2b treatment in CHB patients, we developed a predictive model for HBsAg clearance. The model is as follows:

$$P = \, e^{y} /\left( {1 + e^{y} } \right)$$

$$y = 0.92 \, + \, gender \times \, 0.66 \, - \, HBsAg \, \left( {\log } \right) \, \times 0.96 \, - \, cirrhosis \times \, 0.88$$

(Note: gender takes the value of 1 for females and 0 for males, cirrhosis takes the value of 1 if present, and 0 if absent). We present this predictive model in the form of the nomogram for quantitative prediction of HBsAg clearance in CHB patients after 48 weeks of Peg-IFNα−2b treatment (Fig. 2A). We can calculate the probability of hepatitis B surface antigen clearance based on the dynamic nomogram. For example, a female without cirrhosis, with a baseline HBsAg of 1000 iu/ml, has a total score of 142 (56 + 45 + 41 = 142) calculated based on our prediction model, and the estimated probability of surface antigen clearance based on the score is 26%.

### Predictive model validation

**Fig. 3:**
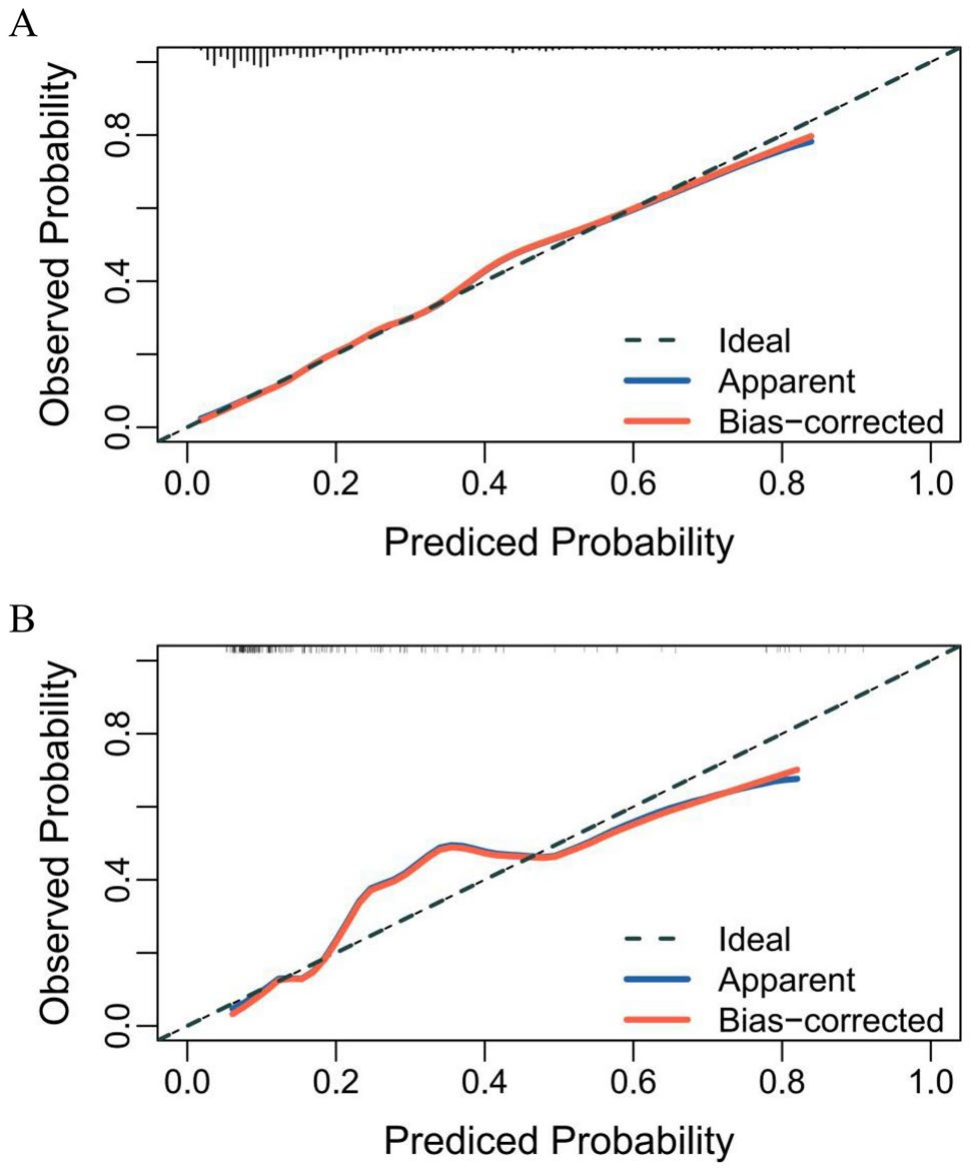
Calibration curves of the predictive models. **A**, **B** The Calibration curves of the predictive function models for the training set (**A**) and validation set (**B**). The y-axis represents the actual probability of seroconversion, while the x-axis represents the predicted probability of seroconversion by the model. The diagonal dashed line signifies the perfect prediction of the ideal model, while the solid line represents the predictive performance of the model

The ROC curve was utilized to evaluate the discriminative capability of the predictive model, with 1-specificity as the x-axis and sensitivity as the y-axis. The ROC curve for the predictive function models and HBsAg of the training and validation sets was plotted. The AUC of the predictive function models for the training and validation sets were 0.802 (95%CI 0.762–0.842) and 0.816 (95%CI 0.733–0.898), The AUC of the HBsAg for the training and validation sets were 0.790 (95%CI 0.750–0.831) and 0.823 (95%CI 0.747–0.899). respectively, and detailed evaluation results are presented in Fig. 2B, C, D, E, indicating a good discriminative ability of the predictive model. Meanwhile, the calibration curve and the Hosmer–Lemeshow test were employed to assess the calibration of the predictive model. The results of the calibration curve (Fig. 3) and the Hosmer–Lemeshow test (*p* = 0.615) both suggest that the predictive model is well-calibrated.

## Discussion

CHB has emerged as a significant global public health concern. Peg-IFN α enhances innate immunity, triggers T-cell-mediated immune responses, suppressing HBV protein synthesis, and reducing the levels of covalently closed circular DNA (cccDNA). This leads to a greater clearance of HBsAg than that achieved with NAs [11]. The clearance of HBsAg in serum is considered indicative of clinical recovery from hepatitis B infection, thus serving as the preferred endpoint for treatment [12]. In this study, the clearance of HBsAg at 48 weeks was used as the endpoint measure. By performing multivariate analysis, we systematically evaluated the factors affecting HBsAg clearance and observed three predictive variables: gender, baseline HBsAg levels, and liver cirrhosis.

### Gender

This study found that, compared to male CHB patients, female patients have a higher chance of HBsAg clearance after 48 weeks of interferon therapy (OR = 1.879, 95% CI 1.2–2.943, *p* = 0.006). This finding is consistent with previous multiple research results. A large cohort study involving 518 HBeAg-negative CHB patients demonstrated that female patients have a 1.93 times higher chance of achieving virologic response at 24 weeks after interferon therapy compared to male patients [13]. Research conducted by Chann et al. on HBeAg-positive CHB patients also demonstrates that females are a favorable population for interferon therapy, exhibiting a higher interferon response rate [14]. However, the precise mechanisms behind the higher interferon response in female patients still require further investigation.

### Baseline HBsAg levels

It has been confirmed that baseline HBsAg levels are a highly influential predictor of HBsAg clearance [15]. A retrospective cohort study demonstrated that lower baseline HBsAg levels are associated with a higher probability of HBsAg clearance after 48 weeks of interferon therapy. Furthermore, baseline HBsAg levels < 100 lU/mL are considered an independent predictive indicator of clinical cure [16]. In long-term NAs therapy, the combination treatment strategy of Peg-IFNα−2b in HBeAg-negative CHB with HBsAg ≤ 1500 IU/mL yields a higher HBsAg clearance rate compared to monotherapy with NAs. Furthermore, lower baseline HBsAg levels, lower HBsAg levels at 12 and 24 weeks of follow-up, and a rapid decline of HBsAg in the early stage of treatment (weeks 12 and 24) are independent predictive factors for HBsAg clearance in the Peg-IFNα−2a combination therapy [17]. Our research findings are consistent with the above statement, showing that the lower the baseline HBsAg level, the higher the probability of HBsAg clearance at 48 weeks (OR = 0.371, 95% CI 0.307–0.448, *p* < 0.001).

### Cirrhosis

This study found that the presence of cirrhosis also influences the probability of HBsAg clearance in interferon-treated patients at 48 weeks. The probability of HBsAg clearance at 48 weeks is significantly lower in patients with baseline cirrhosis (OR = 0.438, 95% CI 0.221–0.868, *p* = 0.018). Prior studies have also indicated that patients with mild liver fibrosis are generally more tolerant to treatment and exhibit a higher response to Peg-IFN therapy compared to those with advanced liver fibrosis or cirrhosis [18]. Due to the numerous adverse reactions associated with interferon itself, patients with cirrhosis or decompensated cirrhosis are usually excluded from study cohorts. Therefore, further research is required to confirm the impact of cirrhosis on interferon response rates.

A number of studies have demonstrated that age is a significant factor influencing the clearance of HBsAg in patients with hepatitis B treated with interferon. These studies have indicated that the likelihood of HBsAg clearance decreases with age [13–15, 19]. Furthermore, patients with low baseline HBV DNA levels [13, 18], HBeAg negativity [20], and ALT elevations exceeding five times the upper limit of normal (5 × ULN) [14] demonstrate enhanced responsiveness to interferon therapy. However, multivariate analyses in this study revealed that age, baseline HBV DNA level, baseline HBeAg and ALT levels were not significantly associated with HBsAg clearance after 48 weeks of interferon therapy. This may be attributed to the characteristics of the study population, differences in follow-up time, sample size limitations, and differences in study endpoints.

Following this, we developed a rigorous probability prediction model for seroconversion through multivariate logistic regression analysis. We identified gender, baseline HBsAg levels, and cirrhosis as key predictors of HBsAg clearance. The model exhibited strong discriminative ability and good fit within both the training and validation datasets. These findings align with those reported by Jiang et al [21], who also highlighted the importance of these factors in predicting HBsAg seroclearance. Moreover, our model’s predictive performance was validated using ROC curve analysis, demonstrating high discriminative ability with an AUC of 0.802 in the training set and 0.816 in the validation set. This strong performance indicates that our model can reliably predict HBsAg clearance in CHB patients undergoing Peg-IFNα−2b therapy. Zhang et al [22] have successfully developed a predictive model based on baseline HBsAg levels to forecast the potential for functional cure in CHB patients treated with PEG-IFNα, further underscoring the significance of baseline HBsAg levels. However, our study diverges from Zhang et al by incorporating additional factors—gender and the presence of cirrhosis—into the model, alongside baseline HBsAg levels. In comparison to previous studies [13, 14, 21, 23, 24], our study has the following advantages: Firstly, the prediction model utilises nomograms, a non-invasive risk prediction model that is crucial for screening and clinical practice [25], and has been extensively employed for risk assessment of numerous diseases [26, 27]. Secondly, our study had a broader coverage, including all patients with CHB aged 18–80 years who received 48 weeks of Peg-IFNα−2b therapy. In conclusion, the sample size of our study is more appropriate and the training and validation sets are more adequate. However, This study did not include certain important influencing factors, such as HBV genotypes. Previous research has demonstrated the association between interferon response and HBV genotypes [13, 28, 29]. But, the absence of these tests improves their applicability to clinical practice, as these tests are often not conducted in clinical settings due to limitations in economic resources and testing capabilities. Moreover, this study was exclusively conducted in China, and the geographical limitations may affect the generalizability of the results to other populations with different genetic backgrounds, healthcare systems, and environmental factors. Nonetheless, being a multicenter study, it still demonstrates strong representativeness among Asian populations.

## Conclusion

In this analysis, three validated predictors, including gender, baseline HBsAg levels, and cirrhosis, are highly significant in predicting the probability of HBsAg clearance in CHB patients after 48 weeks of Peg-IFN-2b therapy.

## Supplementary Information

Below is the link to the electronic supplementary material.

Supplementary file1 (XLSX 10 kb)

Supplementary file2 (XLSX 10 kb)

## Data Availability

The datasets generated or analyzed during the current study are available from the corresponding author on reasonable request.
